# Otopathogenic *Pseudomonas aeruginosa* induces MyD88-dependent auditory hair cell damage

**DOI:** 10.1038/cddiscovery.2016.30

**Published:** 2016-12-05

**Authors:** Rahul Mittal, M’hamed Grati, Denise Yan, Xue Z Liu

**Affiliations:** 1 Department of Otolaryngology, University of Miami Miller School of Medicine, Miami, FL, USA; 2 Department of Biochemistry, Miami, FL, USA; 3 Department of Human Genetics, University of Miami Miller School of Medicine, Miami, FL, USA; 4 Department of Otolaryngology, Xiangya Hospital, Central South University, Changsha, Hunan, China

Dear Editor,

Otitis media (OM) comprises a group of complex inflammatory and infectious disorders affecting the middle ear.^1^ Despite appropriate therapy, acute OM can progress to chronic OM (COM).^2^ One form of COM is chronic suppurative OM (CSOM), which is generally caused by *Pseudomonas aeruginosa* (PA).^3^ The most common sequelae of CSOM is sensorineural hearing loss (SNHL) characterized by auditory hair cell damage.^4^ However, the molecular mechanisms underlying auditory hair cell damage that causes hearing loss during CSOM are not known. We argued that inflammatory mediators generated during PA infection of the middle ear epithelial cells (MEECs) can pass from middle to inner ear and affect the viability of auditory hair cells. To examine this hypothesis, filtered supernatants from 2- and 16-h PA*-*infected human MEECs (HMEECs)^5^ were used to treat the primary organ of Corti explant cultures from 3-day-old rats^6^ for 24 h, and then the explants were subjected to confocal and scanning electron microscopy (SEM). As a control, a supernatant from uninfected HMEECs was used. The samples were stained with fluorescein isothiocyanate phalloidin to visualize hair cells (HCs). The explant cultures treated with supernatant from uninfected HMEECs showed three rows of completely preserved outer (OHCs) and one row of inner (IHCs) hair cells (Figure 1a). In contrast, there was death of OHCs and IHCs in apical, middle and basal turns when explant cultures were treated with PA*-*infected HMEECs supernatants, both at 2- and 16-h (Figure 1a). Quantification of these data also confirmed significant reduction in the number of OHCs and IHCs in all turns of organ of Corti (Figure 1a). Abnormal bundle morphology in the treated explant cultures as compared to the control group was also observed in SEM analyses (Figure 1b). This was reflected by the abnormal fusion of stereocilia, and by severe disruption and splaying of hair bundles. These results suggest that inflammatory mediators generated in response to bacterial invasion of MEECs have the potential to damage auditory HCs leading to SNHL.

The interaction of pathogens with host-pattern recognition receptors, such as Toll-like receptors (TLRs), stimulates the release of inflammatory mediators.^7^ MyD88 is used by almost all TLRs (except TLR3) to start the signaling cascade.^8^ Therefore, to determine whether MyD88 pathway is involved in auditory HC damage, the expression of MyD88 was silenced in HMEECs by transfecting it with MyD88 siRNA and then infecting with bacteria. HMEECs transfected with scrambled siRNA and infected with bacteria served as a positive control group, whereas supernatants from uninfected and untransfected cells served as a negative control. Intriguingly, the explant cultures treated with supernatants from PA-infected HMEECs transfected with MyD88 siRNA or uninfected cells showed little or no damage to OHCs and IHCs (Figure 1c). However, severe damage to HC stereocilia was evident when explants were treated with supernatants transfected with scrambled siRNA. These results suggest that PA-induced auditory HC damage is dependent on the MyD88 pathway.

In summary, this study for the first time demonstrated that interaction of PA with MEECs triggers the generation of inflammatory mediators that causes auditory HC damage, which is dependent on the MyD88 pathway. We believe that this *in vitro* cell culture model can be used to understand the pathogenetic mechanisms employed by otopathogenic PA to cause inner ear damage. This cell culture model will also be of immense significance to understand middle–inner ear interaction during the pathogenesis of OM. Further studies are warranted to characterize the role of MyD88 in CSOM that will open up avenues to design novel treatment modalities against the disease.

## Figures and Tables

**Figure 1:**
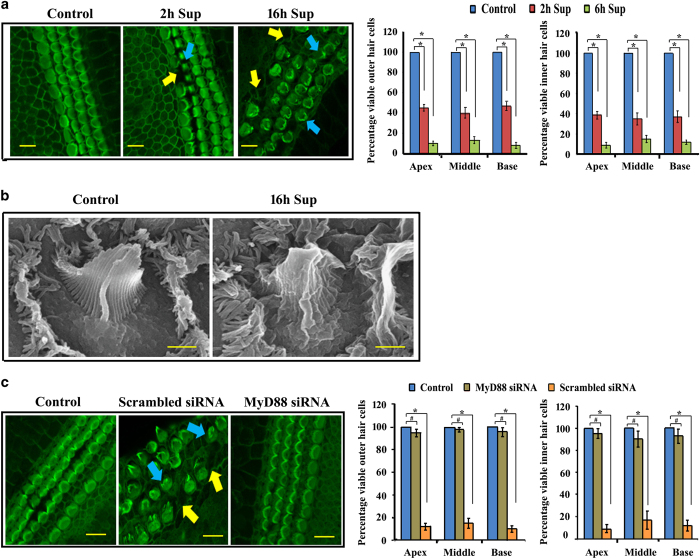
(**a**) Rat organ of Corti explant cultures treated with PA-infected HMEEC supernatants demonstrate severe HC damage and reduction in number compared to control group. Scale bars 10 *μ*m. (**b**) Scanning electron micrograph of organ of Corti treated with PA-infected HMEECs supernatant shows severe hair bundle damage compared with control group. Scale bars 1 *μ*m. (**c**) Rat organ of Corti treated with supernatants from MyD88 siRNA-transfected HMEECs shows no or little HC damage compared with supernatants from scrambled siRNA-transfected cells. Scale bars 10 *μ*m. *P<0.001 or ^#^ *P*>0.05. Yellow arrows indicate missing HCs and blue arrows denote dying HCs.

## References

[bib1] Minovi A , Dazert S . GMS Curr Top Otorhinolaryngol Head Neck Surg 2014; 13: Doc11. 25587371 10.3205/cto000114 PMC4273172

[bib2] Qureishi A , Lee Y , Belfield K , Birchall JP , Daniel M . Infect Drug Resist 2014; 7: 15–24. 24453496 10.2147/IDR.S39637 PMC3894142

[bib3] Mittal R et al. J Med Microbiol 2015; 64: 1103–1116. 26248613 10.1099/jmm.0.000155 PMC4835974

[bib4] Kolo ES , Salisu AD , Yaro AM , Nwaorgu OG . Indian J Otolaryngol Head Neck Surg 2012; 64: 59–62. 23449378 10.1007/s12070-011-0251-5 PMC3244579

[bib5] Mittal R et al. PLoS One 2014, e91885. 24632826 10.1371/journal.pone.0091885 PMC3954863

[bib6] Grati M et al. Hum Mol Genet 2015; 24: 2482–2491. 25601850 10.1093/hmg/ddv009 PMC4383862

[bib7] Akira S , Takeda K , Kaisho T . Nat Immunol 2001; 2: 675–680. 11477402 10.1038/90609

[bib8] Deguine J , Barton GM . F1000Prime Rep 2014; 6: 97. 25580251 10.12703/P6-97 PMC4229726

